# Evolution of 2014/15 H3N2 Influenza Viruses Circulating in US: Consequences for Vaccine Effectiveness and Possible New Pandemic

**DOI:** 10.3389/fmicb.2015.01456

**Published:** 2015-12-22

**Authors:** Veljko Veljkovic, Slobodan Paessler, Sanja Glisic, Jelena Prljic, Vladimir R. Perovic, Nevena Veljkovic, Matthew Scotch

**Affiliations:** ^1^Center for Multidisciplinary Research, Institute of Nuclear Sciences Vinca, University of BelgradeBelgrade, Serbia; ^2^Galveston National Laboratory, Department of Pathology, University of Texas Medical BranchGalveston, TX, USA; ^3^Department of Biomedical Informatics, Arizona State UniversityScottsdale, AZ, USA; ^4^Center for Environmental Security, Biodesign Institute and Global Security Initiative, Arizona State UniversityTempe, AZ, USA

**Keywords:** influenza virus, seasonal influenza vaccine effectiveness, phylogenetic analysis, H3N2, pandemic potential

## Abstract

A key factor in the effectiveness of the seasonal influenza vaccine is its immunological compatibility with the circulating viruses during the season. Here we propose a new bioinformatics approach for analysis of influenza viruses which could be used as an efficient tool for selection of vaccine viruses, assessment of the effectiveness of seasonal influenza vaccines, and prediction of the epidemic/pandemic potential of novel influenza viruses.

## Introduction

Vaccination is the most effective way to prevent infection with seasonal influenza viruses. As a consequence of the high evolutionary rate and antigenic drift of influenza viruses, vaccines are produced annually in preparation of the upcoming season. Generally, the World Health Organization (WHO) recommends the vaccine for the Northern Hemisphere in February and production begins once the FDA approves it (Food and Drug Administration, 2014). Evolution of influenza viruses between the time of vaccine selection and the beginning of the flu season (week 40 for the Northern Hemisphere) can seriously hamper vaccine efficacy. For this reason, in most years, the flu vaccine is 50 to 70% effective (Center for Disease Control and Prevention, 2014a). This is especially a concern for influenza A viruses which evolve more rapidly than influenza B viruses (Nobusawa and Sato, 2006).

For the 2014/15 influenza season, the trivalent United States-licensed influenza vaccine contains the same virus strains as the 2013/14 version: an A/California/7/2009 (H1N1)-like virus, an A/Texas/50/2012 (H3N2)-like virus, and a B/Massachusetts/2/2012-like (Yamagata lineage) virus (Food and Drug Administration, 2014). In contrast to circulating H1N1 and B viruses, which are genetically similar to their vaccine viruses, 52% of H3N2 viruses that circulated during this flu season were antigenically different from the H3N2 vaccine strain (Center for Disease Control and Prevention, 2014b). As a consequence of this mismatch, the efficacy of the vaccine during the 2014/15 flu season was less than 20% (Center for Disease Control and Prevention, 2015a). The situation in Europe was even worse. The efficacy of the influenza vaccine in the United Kingdom (UK; period 1 October 2014 to 16 January 2015) was only 3.4% effective overall and 2.3% specifically against A(H3N2; Pebody et al., 2015). Phylogenetic analysis has shown that the 2014–2015 influenza viruses in the UK belong in the same genetic clade (3C) as vaccine strain A/Texas/50/2012, but to different subgroups (Pebody et al., 2015). However, it is unlikely that this difference alone explains the very low efficacy of the current vaccine since its homology score is > 98% (Pebody et al., 2015).

In general, the main weakness of phylogenetic analyses based on multiple sequence alignment (MSA), is that sequence similarity does not necessarily imply similarity in biological properties. For example, two protein sequences that differ by a single mutation that is lethal for biological function will likely group together in a phylogeny, whereas two proteins that differ in several mutations that do not affect biological functions will be separated. In order to overcome this obstacle and to improve functional sequence analysis, we recently propose a new distance measure based on the informational spectrum method (ISM), representing the virtual spectroscopy method for analysis of protein-protein interactions (Perovic, 2013; Perovic et al., 2013). The ISM analysis of hemagglutinin from influenza A viruses showed that this viral protein encodes a highly conserved region which is specific for each viral subtype (Veljkovic et al., 2009a,b). This region, which is represented by a characteristic frequency in the informational spectrum (IS) of hemagglutinin, determines the virus/receptor interaction (Veljkovic et al., 2009a,b; Schmier et al., 2015) and the immunological cross-reactivity between different viral subtypes (Vergara-Alert et al., 2012).

In this paper, we develop and compare phylogenies based on MSA versus our IS approach for 2014/15 influenza viruses and vaccine strains. Our results identify a potential reason for the very low effectiveness during the 2014/2015 flu season and could also be used to assess vaccine effectiveness for 2015/2016. In addition, the IS analysis of human and animal H3N2 viruses circulating in 2015 in the US revealed a novel swine virus with increased pandemic potential.

## Materials and methods

### Viruses

We analyzed the hemagglutinin HA1 region from (i) 2,379 H3N2 viruses collected in Europe and North America from January 2014 to February 2015, (ii) 804 human and animal H3N2 viruses collected in the US from January to August 2015 (non-redundant sequences are given in Data Sheet 1) that were stored in GISAID (GISAID database^1^ and (iii) vaccine viruses A/Texas/50/2012 and A/Switzerland/9715293/2013 for 2014/2015 and 2015/2016 flu season, respectively.

### Informational spectrum method

The ISM is the virtual spectroscopy method for calculation of the long-range properties of biological macromolecules (Veljkovic et al., 1983). The ISM is based on the electron-ion interaction potential (EIIP) representing molecular descriptor which determines long-range interactions (interactions on distances >5Å) between biological molecules. This parameter is defined by the following equation:

(1) $$\begin{array}{r} EIIP=0.25\frac{Z^{*}\sin\left(1.04\pi Z^{*}\right)}{2\pi } \end{array}$$

where Z^*^ is the average quasivalence number (AQVN) determined by

(2) $$Z^{*}=\frac{1}{N}\sum_{i=1}^{m}n_{i}Z_{i}$$

where *Z*_*i*_ is the valence number of the *i*-th atomic component, *n*_*i*_ is the number of atoms of the *i*-th component, *m* is the number of atomic components in the molecule, and *N* is the total number of atoms. The EIIP values calculated according to equations (1) and (2) are expressed in Rydberg (Ry) units.

The ISM consists of three basic steps:

1. Transformation of alphabetic code of primary protein structure into a sequence of numbers representing EIIP of each component.
2. Conversion of numerical sequence by fast Fourier Transformation into information spectrum, which reveals dominant frequency peaks representing information encoded in the primary structure of protein.
3. Extraction of common information which is encoded in primary structure of proteins by calculation of the Consensus Information Spectrum (CIS).

### The phylogenetic analysis

**Figure 1:**
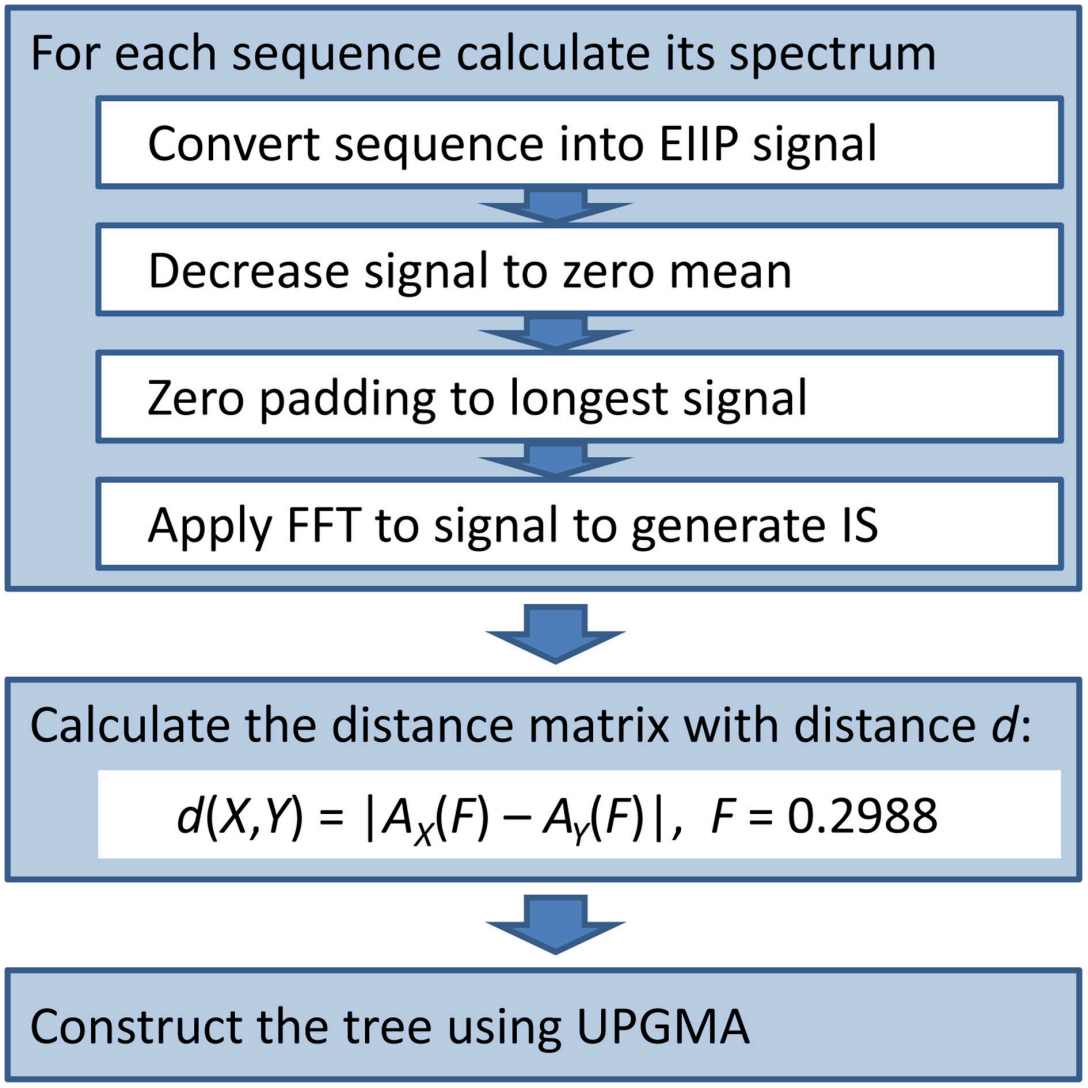
**The schematic presentation of the ISTREE algorithm**.

We used the ISM-based phylogenetic algorithm (ISTREE; Perovic, 2013; Perovic et al., 2013) to generate the phylogenetic tree of the HA1 influenza segment. We previously describe our ISTREE algorithm (Perovic, 2013; Perovic et al., 2013) and in Figure 1, we provide a schematic (For access to ISTREE, we refer the reader to http://istree.bioprotection.org). Here, we used an ISM distance measure *d* defined on the specific frequency *F* = 0.2988. This frequency was extracted by CIS as the common frequency component for novel H3N2 HA1.

For development of the conventional phylogenetic tree based on MSA-based, we used the MEGA5 (Tamura et al., 2011) software package. For the MSA calculation of sequences the MUSCLE algorithm of MEGA5 software was applied.

## Results

**Figure 2:**
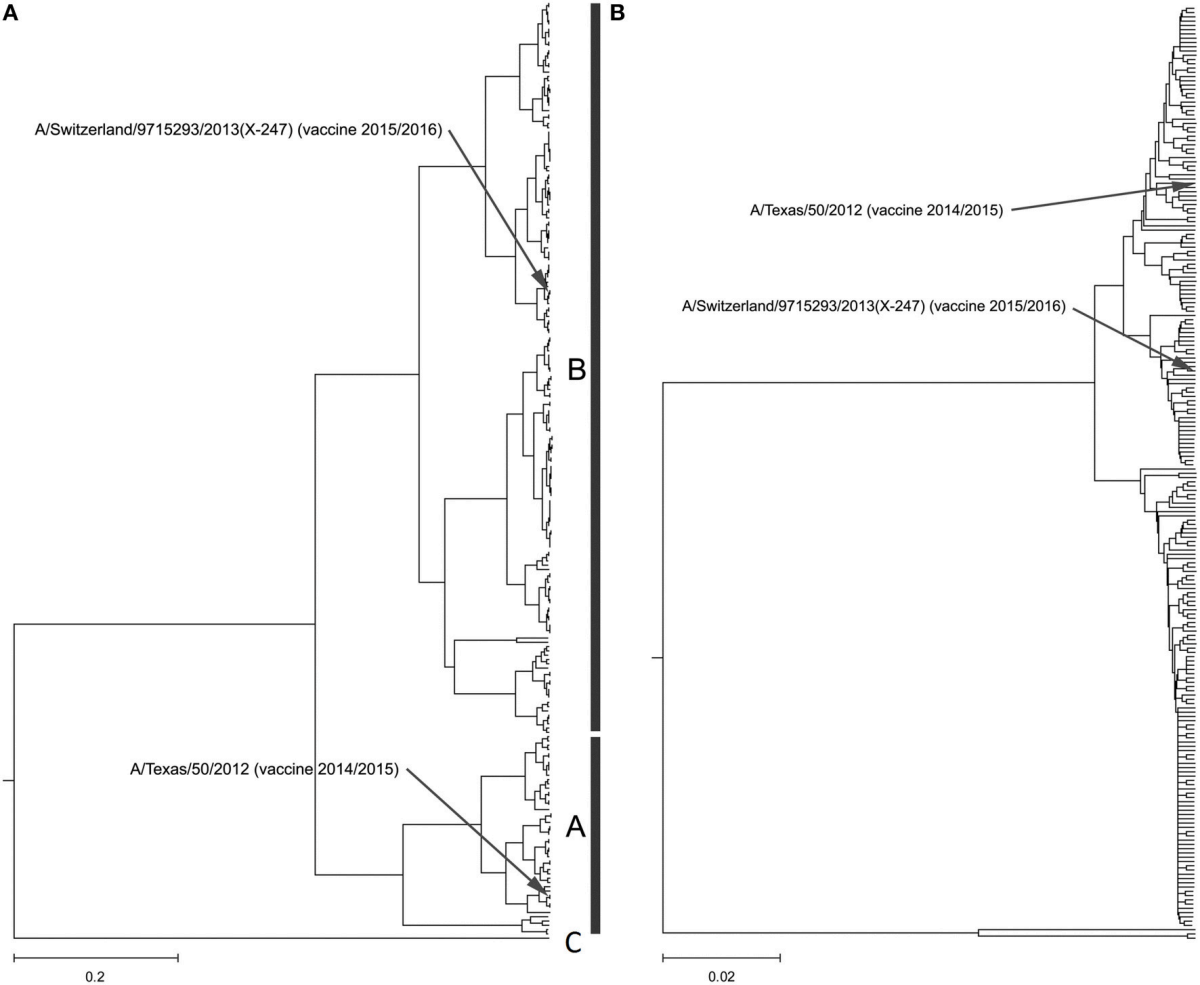
**Phylogenetic analysis of H3N2 viruses collected in North America from January 2014 to February 2015**. **(A)** The ISM-based phylogenetic tree; **(B)** The MSA-based phylogenetic tree.

Our calculation of the CIS for H3N2 HA1 showed that these proteins are characterized with the common biological property represented with the IS frequency *F*_(0.299)_. In Figure 2A and Supplementary Image 2, we present the ISM-based phylogenetic tree for HA1 from H3N2 viruses collected in United States (US) from January 2014 to February 2015 (Data Sheet 2). We constructed this tree using the amplitude on the frequency *F*_(0.299)_ as a distance matrix of the HA1 sequences. As can be seen (Figure 2A and Supplementary Image 2), the viruses formed two separated clusters A and B. This difference is not visible in the MSA-based phylogenetic tree (Figure 2B and Supplementary Image 3).

**Figure 3:**
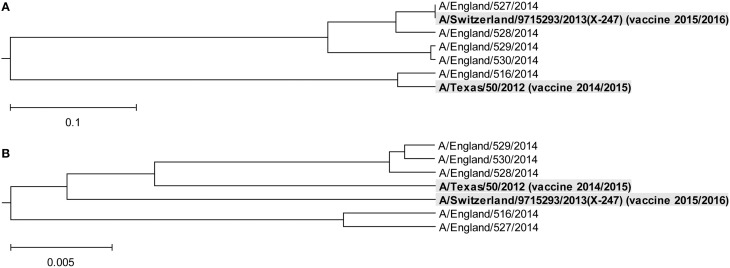
**Phylogenetic analysis of H3N2 viruses collected in England from January 2014 to February 2015**. **(A)** The ISM-based phylogenetic tree; **(B)** The MSA-based phylogenetic tree.

In Figure 3, we show the ISM-based and MSA-based phylogenetic tree for HA1 from viruses collected in UK from January 2014 to February 2015. These viruses also are grouped in two separate clusters A and B. Of note is that that number of viruses in group B is significantly higher than in the group A suggesting that these viruses dominate in the 2014/15 flu season in the US and the UK. According to the IS concept, viruses from group A and B have different interacting and immunological profiles (Vergara-Alert et al., 2012; Perovic, 2013; Perovic et al., 2013). As can be seen in Figures 2A, 3A, H3N2 vaccine strain A/Texas/50/2012 belongs to the smaller group A, and does not match viruses from the dominant group B. It is possible cause for the low vaccine efficacy in this season.

**Figure 4:**
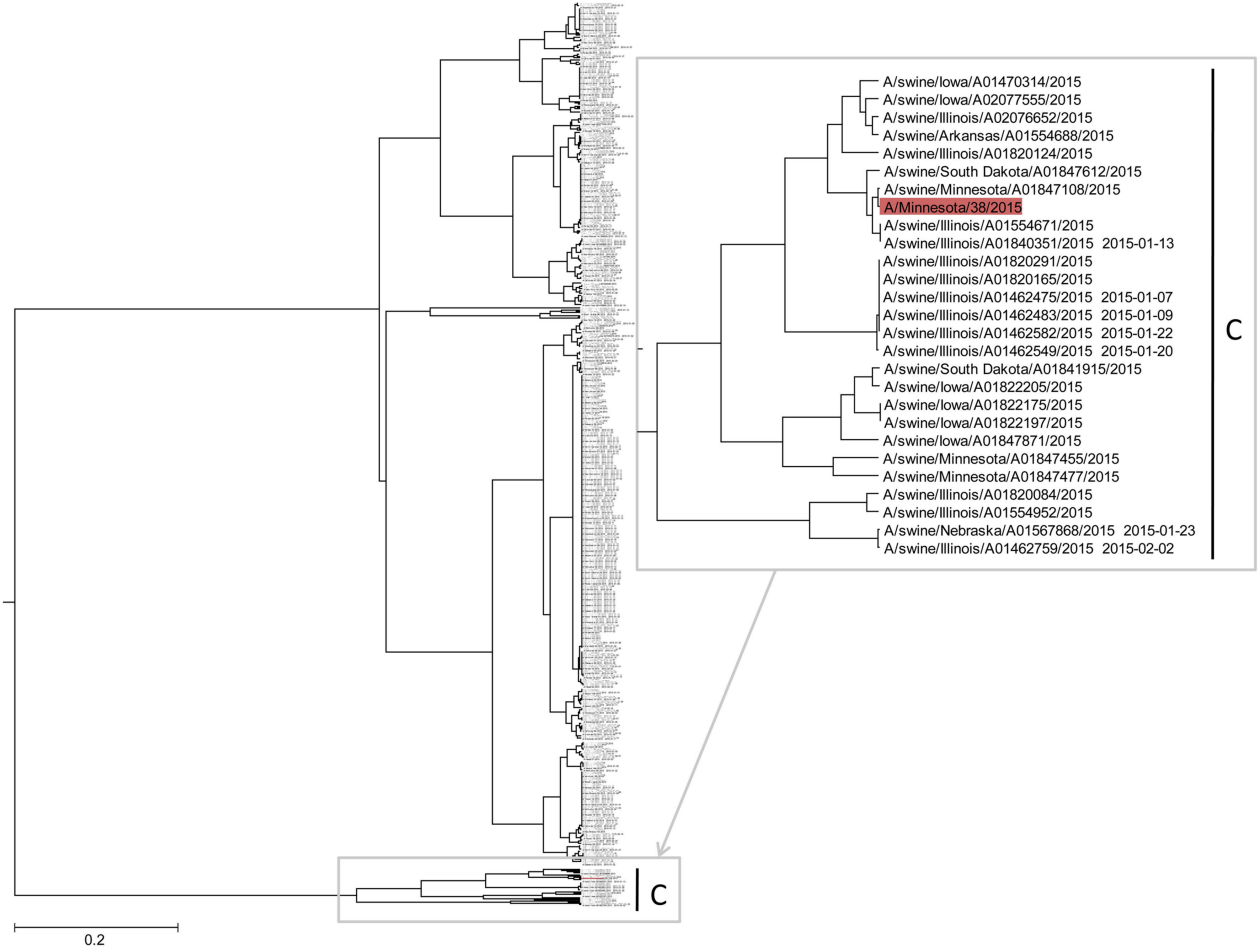
**The ISM-based phylogenetic tree of H3N2 viruses collected in US from January to August 2015**.

In Figure 4 and Supplementary Image 1, we show the ISM-based phylogenetic analysis of H3N2 viruses of animal and human origin isolated in US from January to August 2015. The tree reveals a cluster (group C) of variant human-like swine H3N2 viruses (H3N2v), which are distinct from groups A and B. This result indicates that the current 2015/16 seasonal vaccine will not provide adequate protection against H3N2v in the event of an epidemic.

**Figure 5:**
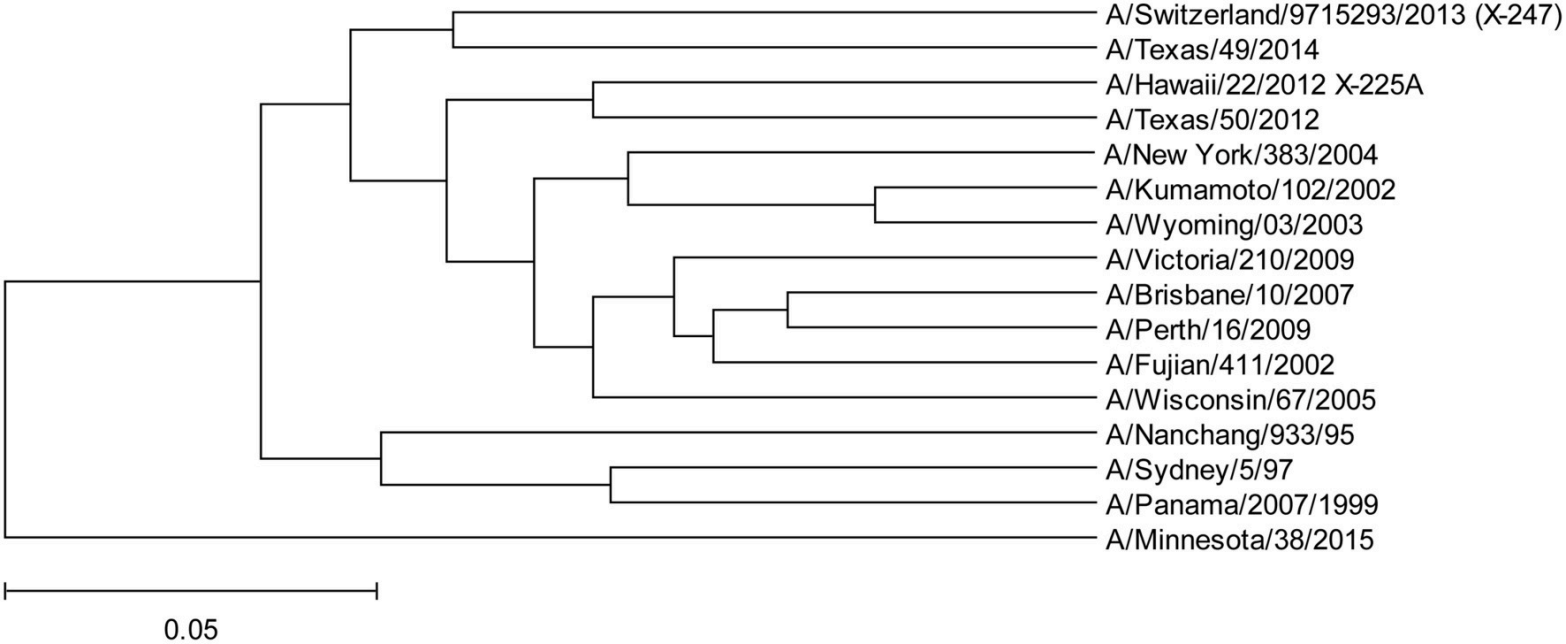
**The Phylogenetic analysis of H3N2 vaccine viruses and human H3N2v A/Minnesota/38/2015**.

To assess protective capacity of current and previous seasonal vaccines against novel H3N2v virus, we compared informational spectrum of HA from human H3N2v (A/Minnesota/38/2015) and vaccine H3N2 viruses used from 1995 to 2015. The results (Figure 5) suggest that not one of the current and previous H3N2 vaccines could give effective protection against H3N2v viruses.

**Figure 6:**
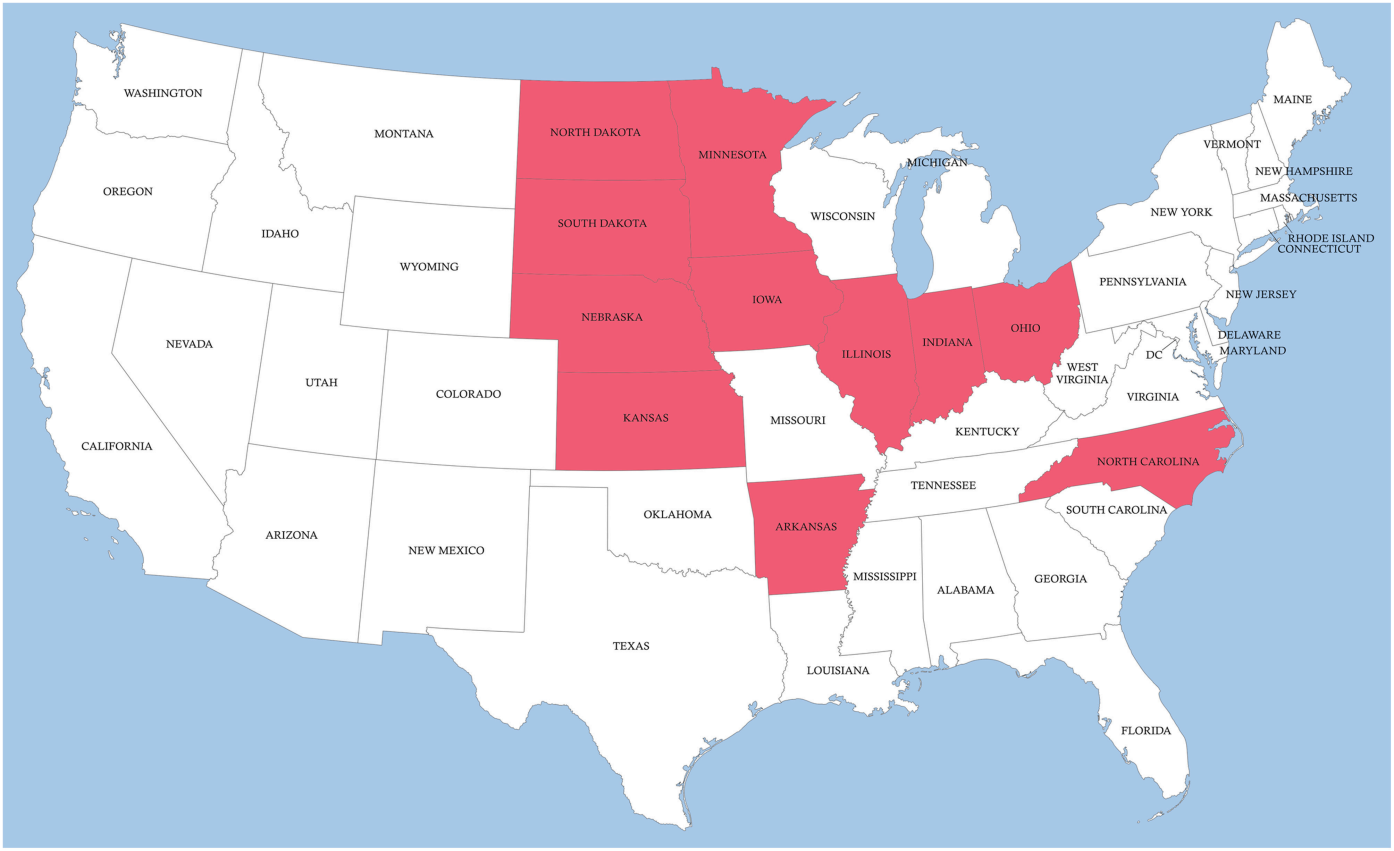
**Distribution of novel H3N2v circulating in 2015 in US**.

Until August 2015 H3N2v viruses are circulating only in the US Middle-East of US and are not present in other parts of the country (Figure 6). Close monitoring of the dynamics of the further spread of H3N2v in US and neighboring countries will be important for assessment of the pandemic potential of this virus.

## Discussion

The MSA-based phylogenetic algorithms are commonly used for analysis of the evolution of viruses. These approaches, although give some useful information about the evolution of influenza viruses, also have some serious drawbacks. The main weaknesses of the MSA-based phylogenetic algorithms for analysis of protein sequences are (i) insensitivity to position of the mutations, (ii) failure to consider deletion within sequence, and (iii) lack of the information about biological effect of mutations (e.g., two protein sequences that differ by a single mutation that is lethal for biological function will likely group together in a phylogeny, whereas two proteins that differ in several mutations that do not affect biological functions will be separated). In order to overcome these drawbacks of the MSA-based phylogenetic analysis of influenza viruses, a novel IS-based algorithm was proposed (Perovic, 2013; Perovic et al., 2013). This phylogenetic algorithm allows (i) assessment of biological effect of each particular mutation and (ii) analysis of the functional evolution of proteins (Perovic, 2013; Perovic et al., 2013; Schmier et al., 2015). Here we develop and compare phylogenesis based on MSA versus ISM for 2014/15 influenza viruses and vaccine strains.

In February 2015, the World Health Organization. (2015) recommended an A/Switzerland/9715293/2013 (H3N2)-like virus as a vaccine strain for the 2015/16 season (16). Our analysis of the HA gene segment from this virus suggests that it belongs to group B of circulating H3N2 viruses (Figures 2, 3) and thus is an appropriate choice for the vaccine.

Since 2011, clinicians and public health professionals discovered H3N2v viruses in the US that had HA segments similar to seasonal H3 strains and a matrix (M) gene similar to 2009 H1N1 pandemic viruses (Nelson et al., 2012; Rajão et al., 2015; Center for Disease Control and Prevention, 2015b). The NA gene of these viruses is of the contemporary human N2 lineage (Rajão et al., 2015). Moreover, the naturally occurring mutations in the H3N2v HA are associated with antigenic divergence from human and swine H3 viruses that circulate in US and from current swine and human vaccine strains (Rajão et al., 2015). These results together with our here presented results for viruses isolated during 2015 in US suggest the fast evolution of novel H3N2v toward viruses with increased pandemic potential.

## Conclusion

Using an ISM-based phylogenetic analysis, we identified two distinct clades for 2014/15 H3N2 seasonal influenza in the US and the UK. We found that the majority of circulating viruses for this season belonged to a clade that is genetically different from the vaccine strain. This result was not observed using conventional phylogenetic approaches thus highlighting the potential for ISM as a mechanism to support vaccination design. Our results also suggest that the influenza vaccine recommended by WHO for season 2015/16 will be effective against circulating H3N2 viruses. Because of fast evolution of influenza viruses it not possible to exclude the possibility that new escape mutants will appear during the 2015/16 season. For this reason, further ISM-based phylogenetic monitoring of influenza viruses will be useful.

Our analysis of variant human-like swine H3N2v viruses which spreads in 2015 across the Middle-East of US indicates pandemic potential of this virus. This analysis also suggests that the seasonal influenza vaccine is not efficient against H3N2v. Further close monitoring of evolution and spread of H3N2v is critical for prevention of a possible pandemic which could be caused by this virus.

## Author contributions

Conceived and designed the study: VV, MS, SP. Developed the analysis tools: VP. Analyzed the data: VV, SG, NV, JP. Wrote the paper: VV, MS, SP.

### Conflict of interest statement

The authors declare that the research was conducted in the absence of any commercial or financial relationships that could be construed as a potential conflict of interest.

## Footnotes

^1^http://platform.gisaid.org.

## Supplementary material

Data Sheet 1 **Non-redundant sequences of human and animal H3N2 viruses collected in the US from January to August 2015 (13)**.

Data Sheet 2 **Non-redundant sequences of H3N2 viruses collected in North America from January 2014 to February 2015**.

Supplementary Image 1 **The ISM-based phylogenetic tree of H3N2 viruses collected in US from January to August 2015 (high resolution)**.

Supplementary Image 2 **The ISM-based phylogenetic tree of H3N2 viruses collected in North America from January 2014 to February 2015 (high resolution)**.

Supplementary Image 3 **The MSA-based phylogenetic tree of H3N2 viruses collected in North America from January 2014 to February 2015 (high resolution)**.
