# Supplementary figures and images for: Evolution of 2014/15 H3N2 Influenza Viruses Circulating in US: Consequences for Vaccine Effectiveness and Possible New Pandemic
Source: Front Microbiol. 2015 Dec 22;6:1456. doi: 10.3389/fmicb.2015.01456 (PMC4686605; doi:10.3389/fmicb.2015.01456)

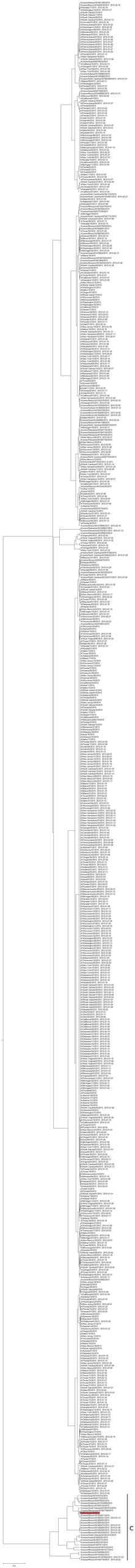

Supplement: Supplementary Image 1 — The ISM-based phylogenetic tree of H3N2 viruses collected in US from January to August 2015 (high resolution). [file Image1.JPEG]

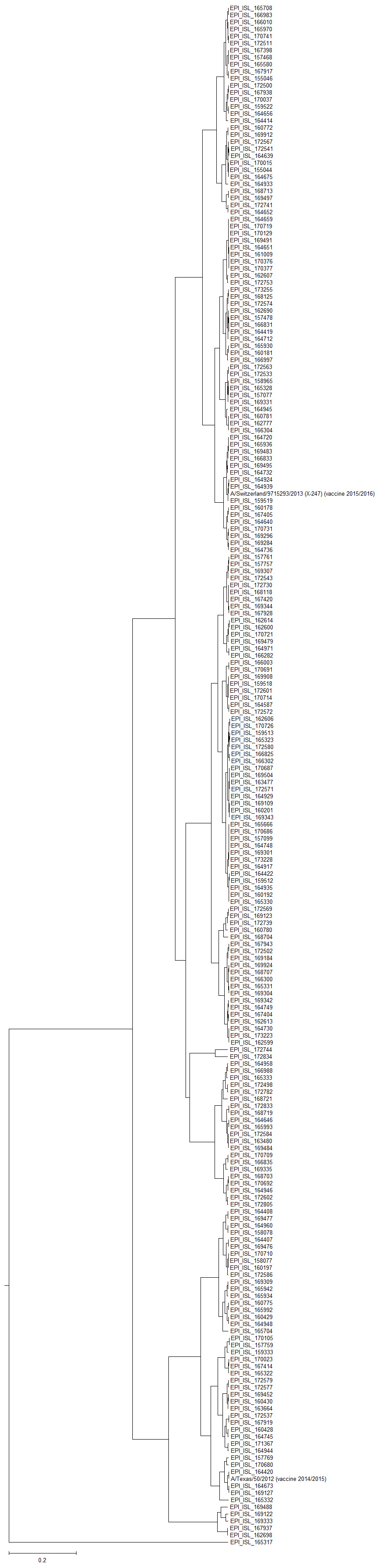

Supplement: Supplementary Image 2 — The ISM-based phylogenetic tree of H3N2 viruses collected in North America from January 2014 to February 2015 (high resolution). [file Image2.JPEG]

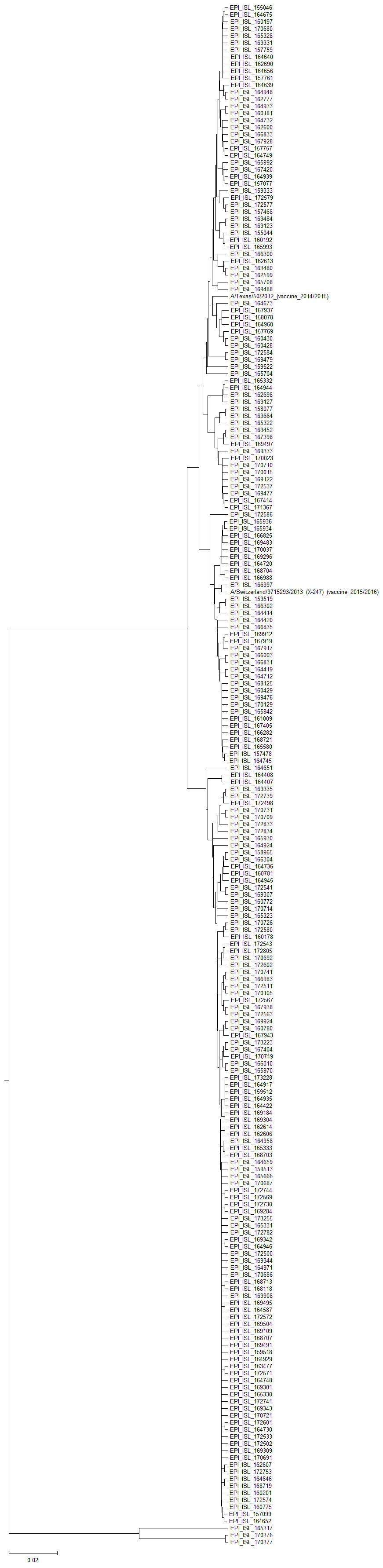

Supplement: Supplementary Image 3 — The MSA-based phylogenetic tree of H3N2 viruses collected in North America from January 2014 to February 2015 (high resolution). [file Image3.JPEG]
